# NDRG2 inhibits hepatocellular carcinoma adhesion, migration and invasion by regulating CD24 expression

**DOI:** 10.1186/1471-2407-11-251

**Published:** 2011-06-16

**Authors:** Jin Zheng, Yan Li, Jiandong Yang, Qiang Liu, Ming Shi, Rui Zhang, Hengjun Shi, Qinyou Ren, Ji Ma, Hang Guo, Yurong Tao, Yan Xue, Ning Jiang, Libo Yao, Wenchao Liu

**Affiliations:** 1 State Key Discipline of Cell Biology, Department of Oncology, Xijing Hospital, The Fourth Military Medical University, Xi'an, 710032, China; 2 Department of Biochemistry and Molecular Biology, The State Key Laboratory of Cancer Biology, The Fourth Military Medical University, Xi'an, 710032, China; 3 Department of Hepatobiliary Surgery, Xijing Hospital, The Fourth Military Medical University, Xi'an, 710032, China; 4 Department of Hematology, Tangdu Hospital, The Fourth Military Medical University, Xi'an, 710038, China; 5 Department of Neurology, Xijing Hospital, The Fourth Military Medical University, Xi'an, 710032, China; 6 Department of Traditional Chinese and Western Medicine of Oncology, Tangdu Hospital, The Fourth Military Medical University, Xi'an, 710038, China

## Abstract

**Background:**

The prognosis of most hepatocellular carcinoma (HCC) patients is poor due to the high metastatic rate of the disease. Understanding the molecular mechanisms underlying HCC metastasis is extremely urgent. The role of CD24 and NDRG2 (N-myc downstream-regulated gene 2), a candidate tumor suppressor gene, has not yet been explored in HCC.

**Methods:**

The mRNA and protein expression of CD24 and NDRG2 was analyzed in MHCC97H, Huh7 and L-02 cells. Changes in cell adhesion, migration and invasion were detected by up- or down-regulating NDRG2 by adenovirus or siRNA. The expression pattern of NDRG2 and CD24 in HCC tissues and the relationship between NDRG2 and HCC clinical features was analyzed by immunohistochemical and western blotting analysis.

**Results:**

NDRG2 expression was negatively correlated with malignancy in HCC. NDRG2 exerted anti-tumor activity by regulating CD24, a molecule that mediates cell-cell interaction, tumor proliferation and adhesion. NDRG2 up-regulation decreased CD24 expression and cell adhesion, migration and invasion. By contrast, NDRG2 down-regulation enhanced CD24 expression and cell adhesion, migration and invasion. Immunohistochemical analysis of 50 human HCC clinical specimens showed a strong correlation between NDRG2 down-regulation and CD24 overexpression (P = 0.04). In addition, increased frequency of NDRG2 down-regulation was observed in patients with elevated AFP serum level (P = 0.006), late TNM stage (P = 0.009), poor differentiation grade (P = 0.002), tumor invasion (P = 0.004) and recurrence (P = 0.024).

**Conclusions:**

Our findings indicate that NDRG2 and CD24 regulate HCC adhesion, migration and invasion. The expression level of NDRG2 is closely related to the clinical features of HCC. Thus, NDRG2 plays an important physiological role in HCC metastasis.

## Background

Invasion and metastasis are closely linked with poor prognosis and death in HCC. Molecules capable of inhibiting invasion and metastasis are attractive candidates for targeted therapy. NDRG2 (GenBank accession no. AF159092), initially identified in our laboratory, belongs to the NDRG (N-myc downstream-regulated genes) family. Members of this gene family are involved in cell growth, differentiation, stress and hormonal responses [1-3]. Recently, NDRG2 has been reported to act as a tumor suppressor [4-6]. In clinical specimens, HCC has low or undetectable levels of NDRG2 compared to normal adjacent tissue. Low expression of NDRG2 is a positive indicator of clinical parameters relevant to metastasis. NDRG2 plays a major role in suppressing HCC metastasis by inhibiting extracellular matrix-based, Rho-driven tumor cell invasion and migration [7,8].

The mechanisms by which NDRG2 inhibits the aggressive behavior of HCC are not fully understood. Adhesion molecules involved in HCC metastasis were screened for possible contribution to NDRG2-mediated tumor inhibition. CD24 was identified as a key NDRG2-regulated gene. CD24 is associated with tumor metastasis [9]. Increased CD24 correlates with aggressive behavior in renal cell carcinoma [10], glioma [11], non-small cell lung cancer [12], breast cancer [13], prostate cancer [14] and ovarian cancer [15,16]. CD24 overexpression is significantly associated with positive nodal status, advanced disease stages and shorter disease-free survival time [17]. CD24 is overexpressed in aggressive HCC cell lines and in the tumor tissues of patients with recurrent HCC. CD24 mRNA overexpression correlates strongly with p53 gene mutation and poor HCC differentiation [18]. Depletion of CD24 decreases cell proliferation and metastasis [19]. In an independent HCC microarray analysis, the prognostic power of CD24 suggested that CD24 may be a putative biomarker for the prediction of early recurrence [20]. Thus, CD24 is a novel molecule involved in HCC tumorigenesis and metastasis.

In this paper, NDRG2 was identified as a regulator of HCC adhesion, migration and invasion. Through adenovirus-mediated NDRG2 overexpression or siRNA-mediated NDRG2 down-regulation in HCC cell lines and immunohistochemistry of HCC clinical specimens, NDRG2 was found to regulate the malignant behaviors of HCC by altering the expression of CD24; moreover, our data suggest that NDRG2 may be a suitable diagnostic marker of HCC.

## Methods

### Cell lines and culture

The human HCC cell lines Huh7 and MHCC97H, and the human liver cell line L-02 were obtained from the Chinese Academy of Sciences (Shanghai, China). Cells were maintained in Dulbecco's Modified Eagle's Medium (DMEM; Invitrogen, Carlsbad, CA, USA) supplemented with 10% fetal bovine serum (FBS; Sijiqing Biological Engineering Materials Co., Hangzhou, China) at 37°C in 95% air and 5% CO_2_.

### Gene infection

A multiplicity of infection (MOI) of 40 was determined experimentally for MHCC97H cells. Cells were seeded in 6-well plates at a density of 5 × 10^5 ^cells/well and incubated to reach approximately 80% confluence. After removing the medium, adenovirus expressing NDRG2 (Ad-NDRG2) or the negative control gene Lac Z (Ad-LacZ) was added in serum-free DMEM, incubated for 2 h, replaced with fresh DMEM supplemented with 10% FBS and incubated for 48 h.

### Gene transfection

Huh7 cells were seeded in 6-well plates at a density of 5 × 10^5 ^cells/well. Cells were transfected with NDRG2 siRNA or negative control siRNA (TaKaRa) using Lipofectamine 2000 (Invitrogen), according to the manufacturer's protocol. NDRG2 siRNA sequences were: 5'-GCUCUCUGGAAAUUCUGAGUUGAUA-3' (sense) and 5'-UUAAGAGCAUAUCUCGCCAGGAUGU-3' (antisense). Negative control siRNA sequences were: 5'-UUCUCCGAACGUGUCACGUTT-3' (sense) and 5'-ACGUGACACGUUCGGAGAATT-3' (antisense). Cells were exposed to siRNA in DMEM for 6 h, after which the medium was replaced with DMEM containing 10% FBS and the cells were incubated for 48 h.

### RNA isolation and Quantitative RT-PCR

Total RNA was isolated from cells using Trizol Reagent (Invitrogen) and quantified. cDNA was synthesized from 5 μg of RNA using AMV reverse transcriptase (Promega, Madison, WI, USA) according to the manufacturer's instructions. The cDNA was used as a template for real-time quantitative PCR using the Prism 7500 real-time PCR instrument (Applied Biosystems Inc., Foster City, CA, USA) and the Universal Mastermix (ABI). Primers were designed using Primer Express Software (ABI). Primer sequences were: NDRG2, 5'-GAGATATGCTCTTAACCACCCG-3' (sense) and 5'-GCTGCCCAATCCATCCAA -3' (antisense), product size: 90 bp; CD24, 5'-ACCTGTTTCCATTCAACAAGAGCAC-3' (sense) and 5'-TCTGAGATCGCACCACTGCAC-3' (antisense), product size: 164 bp; β-actin, 5'-AGCGAGCATCCCCCAAAGTT-3' (sense) and 5'-GGGCACGAAGGCTCATCATT-3' (antisense), product size: 285 bp. The PCR reaction consisted of 12.5 μl of SYBR Green PCR Master Mix, 300 nM each of forward and reverse primers, and 1.5 μl of template cDNA in a total volume of 25 μl. The thermal cycling conditions were as follows: initial denaturation step at 95°C for 30 seconds, followed by 40 cycles of 95°C for 5 seconds and 60°C for 34 seconds. Data were normalized to β-actin which was used as a loading control.

### Western blot analysis

Cells and liver tissues (see below) were lysed in 200 μL of buffer containing 50 mM Tris (pH 7.5), 150 mM NaCl, 1 mM MgCl_2_, 0.5% NP-40, 0.1 mM phenylmethyl sulfonylfluoride (PMSF) and protease inhibitor cocktail. A total of 20 μg of lysate (as measured by BCA protein assay; Pierce, Rockford, IL, USA) was loaded per lane onto 12% SDS polyacrylamide gels for separation by electrophoresis and transfer onto Hybond nitrocellulose membranes (GE Healthcare, Piscataway, NJ, USA). Following transfer, membranes were incubated with 5% fat-free milk in Tris-buffered saline containing 0.05% Tween-20 for 1 h at 37°C. Primary antibody was then added and incubated overnight at 4°C. Primary antibodies were anti-NDRG2 (Abnova, Taiwan, China), anti-CD24 (Santa Cruz Biotechnology, Santa Cruz, CA, USA), and anti-β-actin (Boster, Wuhan, China). After washing three times with PBS, membranes were incubated with a horseradish peroxidase-conjugated goat anti-mouse IgG antibody (Sigma) for 1 h. The blots were developed with chemiluminescence substrate solution (Pierce) and exposed to X-ray film for visualization.

### Adhesion assay

Next, 24-well plates were coated with collagen I (5 μg/cm^2^). Cells exposed to adenovirus or siRNA for 48 h were seeded at a density of 1 × 10^5^/well and then incubated for 80 min. Five duplicate wells were set up for each group. At the end of the experiment, cells were washed twice with PBS to remove non-adherent cells. The remaining cells were counted under a microscope in five randomly-chosen fields per cm^2 ^of substrate surface area. Experiments were repeated three times and data were summarized as mean ± SD.

### Migration assay

Cells (1 × 10^5^) exposed to adenovirus or siRNA for 48 h were plated in 6-well plates and grown to confluence. The monolayer was wounded by scratching with a sterile pipette tip lengthwise along the chamber. After wounding, cells were washed twice with PBS and cultured at 37°C for 24 h. Images were captured immediately after cell wounding (0 h) and 24 h after cell wounding. Wound width (μm) was measured using OpenLab software.

### Invasion assay

*In vitro *invasion assays were performed using 24-well transwell units with Matrigel-coated polycarbonate filters (Corning Costar, Cambridge, MA). Cells exposed to adenovirus or siRNA for 48 h were seeded in the upper chamber of the transwell at 1 × 10^5 ^cells in 500 μl of serum-free medium, while the bottom chamber was filled with 200 μl of medium containing 10% FBS; After 24 h incubation, transwells were fixed with methanol for 15 min and stained with gentian violet for 10 min. Cells in the upper chamber were removed using a cotton swab and cells that invaded through the Matrigel to the other side of the filter were manually counted. Experiments were performed in triplicate. Data represent the average number of cells from three filters.

### HCC clinical specimen preparation

From 2007 to 2010, 50 patients (34 men and 16 women from 21 to 72 years of age) with primary HCC were enrolled in this study at the Xijing Hospital of the fourth military medical university. Tumors were resected and primary HCC was confirmed by a pathologist. The study was approved by the research ethics committee of Xijing Hospital. Proteins were extracted with standard techniques as soon as the liver samples were excised. All patients were prospectively monitored using the α-fetoprotein (AFP) assay. Tumor differentiation was classified according to the Edmondson grading system, with slight modification [21,22], into two groups: well-differentiated HCC (grades I and II) and poorly-differentiated HCC (grades III and IV). NDRG2 and CD24 expression was scored as positive if > 10% of the cells showed moderate to strong staining. Expression was scored as weak if either cytoplasmic or membranous staining was noted in < 10% of the cells. Expression was scored as negative if neither cytoplasmic nor membranous staining was observed [7].

### Immunohistochemical analysis

Four micrometer-thick tissue sections were subjected to immunofluorescent staining analysis. Free-floating liver sections were blocked with 5% normal goat serum in PBS containing 0.3% Triton X-100 for 1 h at room temperature. The sections were then incubated with the following primary antibodies overnight at 4°C: mouse anti-NDRG2 (1:200) or rabbit anti-CD24 (1:200). For double immunofluorescent staining, two antibodies were added at the same time. After incubation with species-specific secondary antibodies conjugated to Cy2 or Cy3 (1:400, Jackson ImmunoResearch, West Grove, PA) for 3 h at room temperature, the fluorescent signals were visualized using a confocal laser microscope (Olympus, Center Valley, PA). NDRG2+ and/or CD24+ immunoreactive areas were obtained bilaterally from every fifth section in a random square unit (125 × 125 μm) and the percentage of immunoreactive area to the total area was calculated. NDRG2/CD24 double-labeled cells were counted manually. Cy2 and Cy3 relative fluorescent intensity was measured using the NIH image-J software.

### Statistical analyses

Statistical analyses were performed using SPSS 11.0 software. Data were summarized as mean ± SD. The χ^2 ^test, one-way ANOVA and post hoc Bonferroni test were used for comparison between groups. P < 0.05 was considered statistically significant.

## Results

### NDRG2 and CD24 expression in HCC and normal liver cells

**Figure 1:**
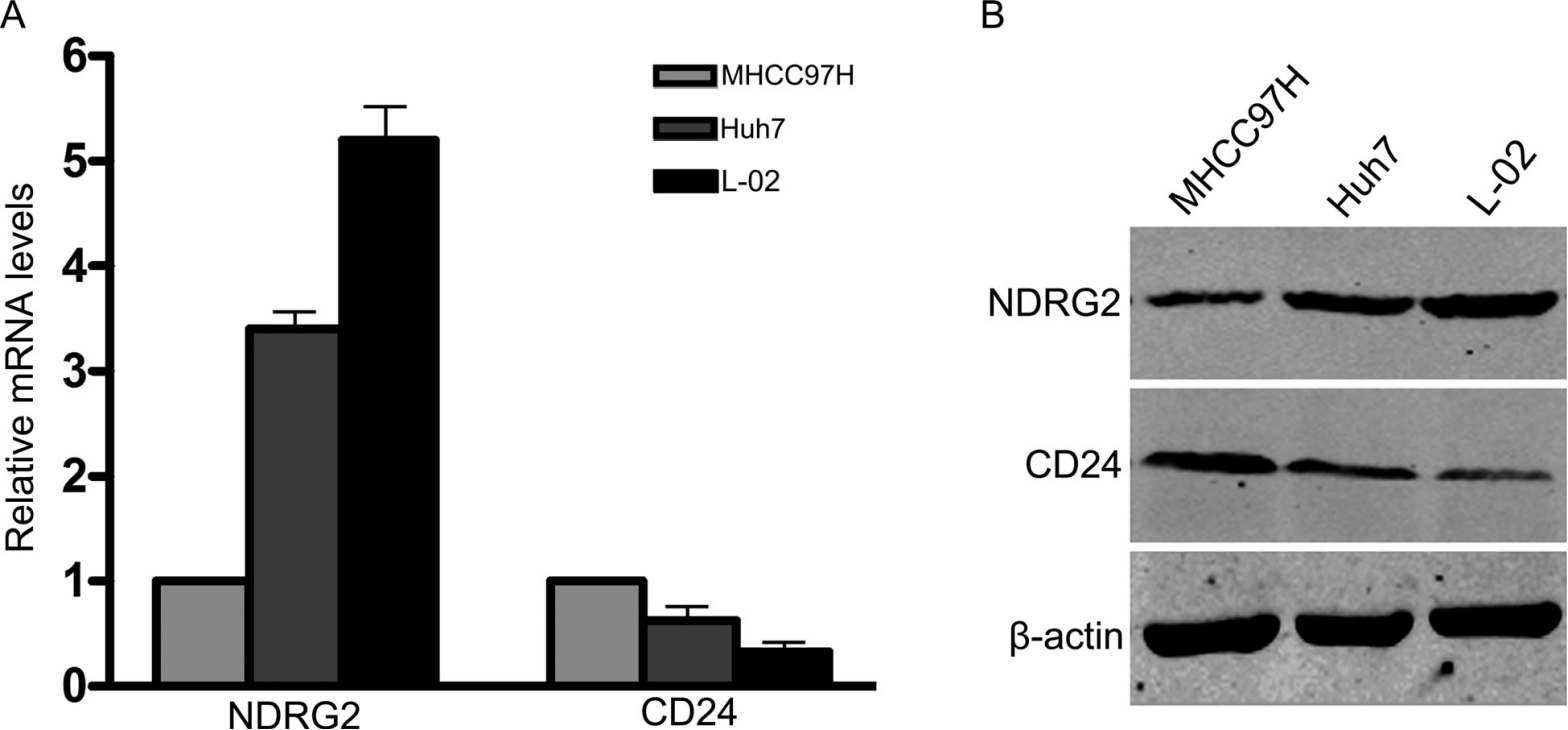
**NDRG2 and CD24 expression in MHCC97H, Huh7 and L-02 cells**. Adherent MHCC97H, Huh7 and L-02 cells were collected. RNA and protein were extracted for the detection of NDRG2 and CD24 expression by qRT-PCR (A) and western blotting analyses (B), respectively. Data represent three independent experiments.

The expression of NDRG2 and CD24 was examined in three liver cell lines. MHCC97H and Huh7 are liver cancer cells. A preliminary experiment indicated that MHCC97H was highly invasive while Huh7 was weakly invasive (data not shown). L-02 is a normal, non-invasive human liver cell line. Results showed that the mRNA and protein expression of NDRG2 in MHCC97H cells was lower than in Huh7 cells. L-02 cells showed the highest level of NDRG2 among the three cell lines. CD24 expression was higher in MHCC97H cells compared to Huh7 cells while L-02 expressed the lowest level of CD24 (Figure 1).

### NDRG2 regulates CD24 expression in HCC cells

**Figure 2:**
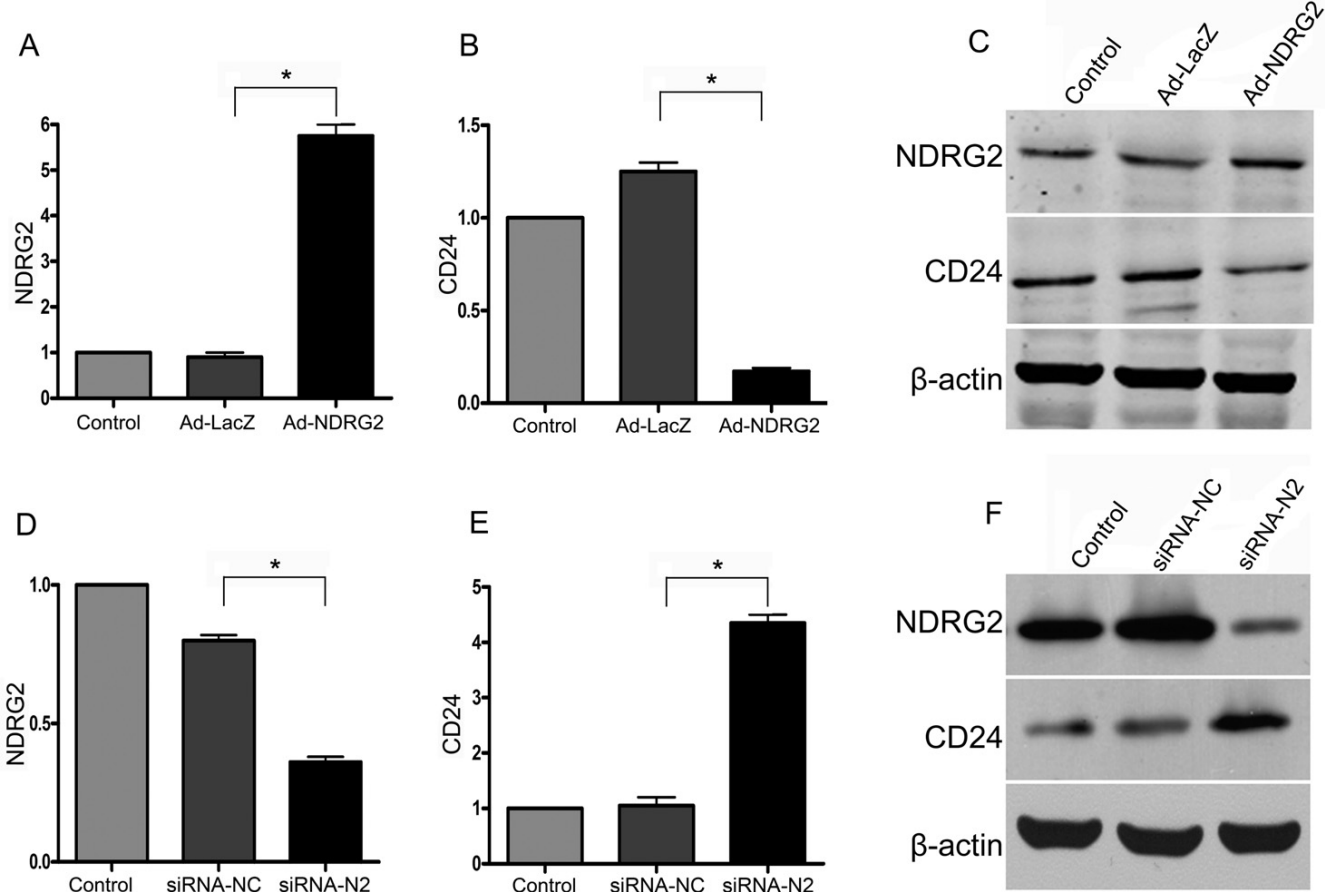
**NDRG2 modulates CD24 expression in HCC cells**. NDRG2-low HCC MHCC97H cells were infected with adenovirus expressing NDRG2 (Ad-NDRG2) or the negative control gene Lac Z (Ad-LacZ) (A-C), while NDRG2-high HCC Huh7 cells were transfected with NDRG2 siRNA (siRNA-N2) or negative control siRNA (siRNA-NC) using Lipofectamine 2000 (D-F). RNA and protein were extracted and subjected to qRT-PCR (A, B, D and E) and western blotting (C and F), respectively. β-actin was used as a loading control. Data represent three independent experiments. *P < 0.05 between groups.

To understand the regulation of NDRG2 and CD24, MHCC97H cells, which express a low level of NDRG2, were transiently infected with adenoviruses expressing NDRG2. Increased NDRG2 mRNA and protein expression was detected in these cells while expression of CD24 mRNA and protein was suppressed (Figure 2A-C). By contrast, transfection of NDRG2 siRNA into NDRG2-positive Huh7 cells increased CD24 expression (Figure 2D-F).

### NDRG2 modulates the adhesion, migration and invasion of HCC cells

**Figure 3:**
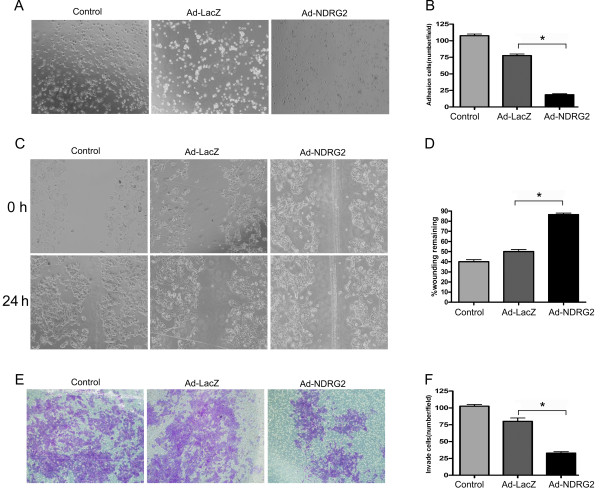
**Overexpression of NDRG2 suppresses the adhesion, migration and invasion of MHCC97H cells**. NDRG2-low HCC MHCC97H cells were infected with adenovirus expressing NDRG2 (Ad-NDRG2) or the negative control gene Lac Z (Ad-LacZ). The cells were subjected to adhesion (A and B), migration (C and D) and invasion assays (E and F), respectively. In (B), (D) and (F), the histograms represent the quantification of cells attached, wounding remaining width and cells having invaded through Matrigel. Data represent three independent experiments. *P < 0.05 between groups.

**Figure 4:**
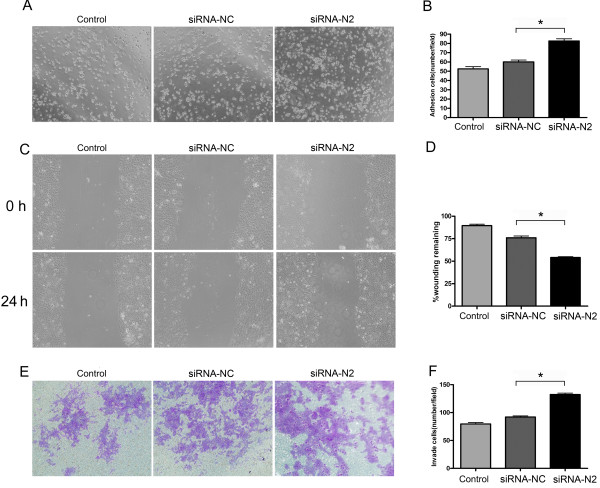
**Down-regulation of NDRG2 increases the adhesion, migration and invasion of Huh7 cells**. NDRG2-high HCC Huh7 cells were transfected with NDRG2 siRNA (siRNA-N2) or the negative control siRNA (siRNA-NC) using Lipofectamine 2000. The cells were subjected to adhesion (A and B), migration (C and D) and invasion assays (E and F), respectively. In (B), (D) and (F), the histograms represent the quantification of cells attached, wounding remaining width and cells having invaded through Matrigel. Data represent three independent experiments. *P < 0.05 between groups.

The behavior of Ad-NDRG2-infected MHCC97H cells was assessed. Restoration of NDRG2 expression significantly inhibited cell adhesion, migration and invasion (Figure 3); By contrast, siRNA-treated Huh7 cells showed increased adhesion, migration and invasion compared to control cells (Figure 4).

### NDRG2 and CD24 show a different expression pattern in HCC clinical specimens

**Figure 5:**
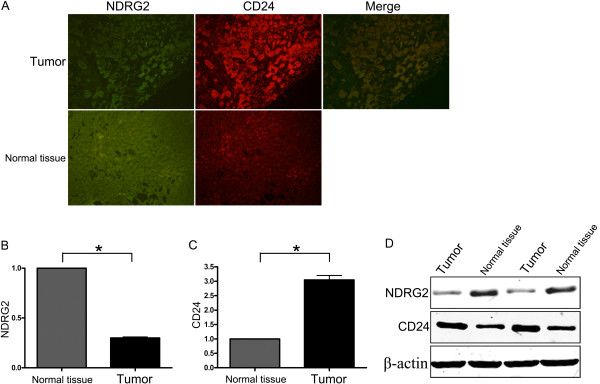
**Analysis of NDRG2 and CD24 expression in HCC clinical specimens**. (A) Tumor tissues showed weak NDRG2 staining and strong CD24 staining whereas normal adjacent tissues showed strong NDRG2 staining and weak CD24 staining. NDRG2 and CD24 colocalized mainly in the cytoplasm. (B) Immunofluorescence quantification with NIH image-J software. The relative fluorescence intensity of NDRG2 and CD24 in tumors and normal adjacent tissues is shown. (C) Representative image of NDRG2 and CD24 expression in tumor and normal adjacent tissue detected by western blotting. *P < 0.05 between groups.

Since CD24 appeared to be regulated by NDRG2 in HCC cell lines, the expression of NDRG2 and CD24 was studied in HCC clinical specimens by indirect immunofluorescence. Double NDRG2/CD24 immunostaining indicated that CD24 was highly expressed in tumors compared to normal adjacent tissues. Decreased NDRG2 expression was detected in tumors while increased expression was detected in normal adjacent tissues. Co-expression of NDRG2 and CD24 was observed in the cytoplasm (Figure 5A). NDRG2 fluorescence intensity was significantly lower in tumors than in normal adjacent tissues (Figure 5B). By contrast, CD24 fluorescence intensity in tumors was higher than in normal adjacent tissues (Figure 5C). To confirm these results, proteins extracted from liver tissues were detected by western blotting analysis. Data showed that NDRG2 expression was decreased in tumor tissues compared to normal adjacent tissues; however, CD24 was enhanced in tumor tissues (Figure 5D).

### Low NDRG2 expression correlates with high CD24 expression in HCC and with histopathological features

HCC with low NDRG2 expression was strongly associated with CD24 overexpression in tumor tissues (P = 0.04). Low NDRG2 level was more frequent in sera with AFP > 320 ng/ml (P = 0.006). Furthermore, a significant negative relationship was observed between NDRG2 and Edmondson's histological grade (P = 0.002), TNM stage (P = 0.009), invasive tumor features such as tumor recurrence (P = 0.024) and tumor invasion (P = 0.004). NDRG2 expression did not correlate with patient age, sex or tumor size (Table 1).

**Table 1:** Correlation of NDRG2 expression with HCC clinical features in 50 primary HCC specimens

Features*		NDRG2 expression Negative/weak (%)	P
Age(years)			
≤50	n = 21	18(85.7)	0.616
> 50	n = 29	22(75.9)	
			
Sex			
Male	n = 34	15(44.1)	0.981
Female	n = 16	7(43.8)	
			
Tumor size			
≤5 cm	n = 39	23(59.0)	0.184
>5 cm	n = 11	4(36.4)	
			
CD24			
Positive	n = 29	22(75.9)	0.040
Negative/weak	n = 21	10(47.6)	
			
Serum AFP level(ng/ml)			
≥ 320	n = 32	25(78.1)	0.006
< 320	n = 18	7(38.9)	
			
TNM stage			
I-II	n = 27	10(37.0)	0.009
III-IV	n = 23	17(73.9)	
			
Edmondson			
I-II	n = 24	7(29.2)	0.002
III-IV	n = 26	19(73.1)	
			
Tumor invasion			
+	n = 32	24(75.0)	0.004
-	n = 18	6(33.3)	
			
Tumor recurrence			
+	n = 33	27(81.8)	0.024
-	n = 12	5(41.7)	

* +, positive; -, negative; n, number of cases; grades I-II, well-differentiated HCC; grades III-IV, poorly differentiated HCC.

## Discussion

NDRG2 antagonizes transforming growth factor-β1 (TGF-β1)-mediated tumor cell invasion by down-regulating the expression of matrix metalloproteinase 2 (MMP2), plasminogen activator inhibitor type 1 (PAI-1) and Rho GTPase activity [7]. The role of TGF-β1 in tumors is not fully understood. TGF-β can both positively and negatively regulate tumor development. Although TGF-β can promote tumor invasion via induction of epithelial to mesenchymal transition (EMT) during the later stages of tumor progression, it is a tumor suppressor during early tumor progression [23]. Thus, the inhibitory role of NDRG2 in HCC may depend on other molecules that have not been fully explored.

In the present study, the expression level of NDRG2 was shown to correlate negatively with HCC invasion and recurrence. In addition, enhanced NDRG2 expression by adenovirus decreased the invasion of HCC cells, while siRNA-mediated inhibition of NDRG2 expression promoted the aggressive behavior of HCC cells. Moreover, NDRG2 suppressed HCC cell adhesion, migration and invasion partly through inhibiting CD24 expression. CD24 mRNA and protein were decreased when HCC cells were infected by Ad-NDRG2. By contrast, CD24 level was increased when HCC cells were transfected with NDRG2 siRNA.

CD24 was first described as a cell surface mucin-like adhesion molecule in hematopoiesis. It is a small heavily glycosylated protein core and consists of 27 amino acids that binds to cell membrane [24]. CD24 has been identified as a ligand for P-selectin, an adhesion receptor on activated platelets and endothelial cells [25]. During metastasis, tumor cells pass through the blood stream by binding to platelets or to endothelial cells via the interaction between CD24 and P-selectin [26]. CD24 increases tumor cell proliferation and adhesion to fibronectin, collagen I, IV and laminin through the activation of alpha3beta1 and alpha4beta1 integrin activity [27]. Thus, CD24 is a regulator of cell-cell and cell-matrix interactions.

CD24 is highly expressed in many human cancers [28]. Immunohistochemical cytoplasmic CD24 staining has a strong prognostic value. CD24 staining intensity in gastric, breast, colon, gallbladder and ovarian cancer correlate with lymph node metastasis [17].

To further explore the expression pattern and relationship of NDRG2 and CD24 in HCC, NDRG2-specific and CD24-specific monoclonal antibodies were used to stain clinical specimens. NDRG2 and CD24 staining was scored semiquantitatively. Higher scores of cytoplasmic CD24 were observed in tumor tissues compared to normal adjacent tissues. Significantly-reduced NDRG2 cytoplasmic staining was detected in tumor tissues compared to normal adjacent tissues. These results, combined with the data from HCC cell lines, indicate that NDRG2 regulates CD24 expression and may affect malignant behavior both *in vitro *and *in vivo*. Furthermore, low NDRG2 correlates strongly with high CD24 and with elevated AFP, TNM, Edmondson stage, HCC invasion and recurrence. Therefore, the low expression of NDRG2 and high expression of CD24 appear to be a common event in HCC and may serve as a prognostic biomarker for malignant transformation in hepatocytes. In addition, NDRG2 may act as a tumor suppressor by regulating different molecules, such as TGF-β1 and CD24, which might lead to greater inhibition of HCC.

## Conclusion

In conclusion, this study shows for the first time that NDRG2 is involved in HCC metastasis through regulation of CD24 expression. This observation broadens our understanding of the molecular mechanisms of HCC metastasis and may lead to the development of new therapeutic approaches. Further studies are needed to explore the pathway through which NDRG2 regulates CD24 and affects the metastasis of HCC.

## Competing interests

The authors declare that they have no competing interests.

## Authors' contributions

JZ, YL, JY carried out the molecular genetic studies, participated in the sequence alignment and drafted the manuscript. QL, MS carried out the immunoassays. RZ, HS, QR, JM participated in the sequence alignment. HG, YT, YX, NJ participated in the design of the study and performed the statistical analysis. LY, WL conceived of the study, participated in its design and coordination and helped to draft the manuscript. All authors read and approved the final manuscript.

## Acknowledgements

This work was supported by the National Natural Science Foundation of China Grants 81072973; 30973437; 30830054; 31070681.

## Pre-publication history

The pre-publication history for this paper can be accessed here:

http://www.biomedcentral.com/1471-2407/11/251/prepub

## References

[B1] Wang L Liu N Yao L Li F Zhang J Deng Y Liu J Ji S Yang A Han H Zhang Y Zhang J Han W Liu X NDRG2 is a new HIF-1 target gene necessary for hypoxia-induced apoptosis in A549 cells Cell Physiol Biochem 2008 21 239 250 10.1159/000113765 18209490

[B2] Shen L Zhao ZY Wang YZ Ji SP Liu XP Liu XW Che HL Lin W Li X Zhang J Yao LB Immunohistochemical detection of Ndrg2 in the mouse nervous system Neuroreport 2008 19 927 931 10.1097/WNR.0b013e32830163d0 18520995

[B3] Boulkroun S Fay M Zennaro MC Escoubet B Jaisser F Blot-Chabaud M Farman N Courtois-Coutry N Characterization of rat NDRG2 (N-Myc downstream regulated gene 2), a novel early mineralocorticoid-specific induced gene J Biol Chem 2002 277 31506 31515 10.1074/jbc.M200272200 12072429

[B4] Shon SK Kim A Kim JY Kim KI Yang Y Lim JS Bone morphogenetic protein-4 induced by NDRG2 expression inhibits MMP-9 activity in breast cancer cells Biochem Biophys Res Commun 2009 385 198 203 10.1016/j.bbrc.2009.05.038 19450561

[B5] Hu XL Liu XP Lin SX Deng YC Liu N Li X Yao LB NDRG2 expression and mutation in human liver and pancreatic cancers World J Gastroenterol 2004 10 3518 3521 15526377 10.3748/wjg.v10.i23.3518 PMC4576239

[B6] Liu N Wang L Liu X Yang Q Zhang J Zhang W Wu Y Shen L Zhang Y Yang A Han H Zhang J Yao L Promoter methylation, mutation, and genomic deletion are involved in the decreased NDRG2 expression levels in several cancer cell lines Biochem Biophys Res Commun 2007 358 164 169 10.1016/j.bbrc.2007.04.089 17470364

[B7] Lee DC Kang YK Kim WH Jang YJ Kim DJ Park IY Sohn BH Sohn HA Lee HG Lim JS Kim JW Song EY Kim DM Lee MN Oh GT Kim SJ Park KC Yoo HS Choi JY Yeom YI Functional and clinical evidence for NDRG2 as a candidate suppressor of liver cancer metastasis Cancer Res 2008 68 4210 4220 10.1158/0008-5472.CAN-07-5040 18519680

[B8] Wu GQ Liu XP Wang LF Zhang WH Zhang J Li KZ Dou KF Zhang XF Yao LB Induction of apoptosis of HepG2 cells by NDRG2 Xi Bao Yu Fen Zi Mian Yi Xue Za Zhi 2003 19 357 360 15163384

[B9] Aigner S Ramos CL Hafezi-Moghadam A Lawrence MB Friederichs J Altevogt P Ley K CD24 mediates rolling of breast carcinoma cells on P-selectin FASEB J 1998 12 1241 1251 9737727 10.1096/fasebj.12.12.1241

[B10] Droz D Zachar D Charbit L Gogusev J Chrétein Y Iris L Expression of the human nephron differentiation molecules in renal cell carcinomas Am J Pathol 1990 137 895 905 1699423 PMC1877557

[B11] Senner V Sturm A Baur I Schrell UH Distel L Paulus W CD24 promotes invasion of glioma cells in vivo J Neuropathol Exp Neurol 1999 58 795 802 10.1097/00005072-199908000-00002 10446804

[B12] Kristiansen G Schluns K Yonwei Y Denkert C Dietel M Petersen I CD24 is an independent prognostic marker of survival in non-small cell lung cancer patients Br J Cancer 2003 88 231 235 10.1038/sj.bjc.6600702 12610508 PMC2377041

[B13] Kristiansen G Winzer KJ Mayordomo E Bellach J Schluns K Denkert C Dahl E Pilarsky C Altevogt P Guski H Dietel M CD24 expression is a new prognostic marker in breast cancer Clin Cancer Res 2003 9 4906 4913 14581365

[B14] Kristiansen G Pilarsky C Pervan J Sturzebecher B Stephan C Jung K Leoning S Rosenthal A Dietel M CD24 expression is a significant predictor of PSA relapse and poor prognosis in low grade or organ confined prostate cancer Prostate 2004 58 183 192 10.1002/pros.10324 14716744

[B15] Kristiansen G Denkert C Schluns K Dahl E Pilarsky C Hauptmann S CD24 is expressed in ovarian cancer and is a new independent prognostic marker of patient survival Am J Pathol 2002 161 1215 1221 10.1016/S0002-9440(10)64398-2 12368195 PMC1867310

[B16] Welsh JB Zarrinkar PP Sapinoso LM Kern SG Behling CA Monk BJ Lockhart DJ Burger RA Hampton GM Analysis of gene expression profiles in normal and neoplastic ovarian tissue samples identifies candidate molecular markers of epithelial ovarian cancer Proc Natl Acad Sci USA 2001 98 1176 1181 10.1073/pnas.98.3.1176 11158614 PMC14728

[B17] Lim SC Oh SH The role of CD24 in various human epithelial neoplasias Pathol Res Pract 2005 201 479 486 10.1016/j.prp.2005.05.004 16164042

[B18] Huang LR Hsu HC Cloning and expression of CD24 gene in human hepatocellular carcinoma: a potential early tumor marker gene correlates with p53 mutation and tumor differentiation Cancer Res 1995 55 4717 4721 7553654

[B19] Yang XR Xu Y Yu B Zhou J Li JC Qiu SJ Shi YH Wang XY Dai Z Shi GM Wu B Wu LM Yang GH Zhang BH Qin WX Fan J CD24 is a novel predictor for poor prognosis of hepatocellular carcinoma after surgery Clin Cancer Res 2009 15 5518 5527 10.1158/1078-0432.CCR-09-0151 19706825

[B20] Woo HG Park ES Cheon JH Kim JH Lee JS Park BJ Kim W Park SC Chung YJ Kim BG Yoon JH Lee HS Kim CY Yi NJ Suh KS Lee KU Chu IS Roskams T Thorgeirsson SS Kim YJ Gene expression-based recurrence prediction of hepatitis B virus-related human hepatocellular carcinoma Clin Cancer Res 2008 14 2056 2064 10.1158/1078-0432.CCR-07-1473 18381945

[B21] Hsu HC Su IJ Lai MY Chen DS Chang MH Chuang SM Sung JL Biologic and prognostic significance of hepatocyte hepatitis B core antigen expressions in the natural course of chronic hepatitis B virus infection J Hepatol 1987 50 45 50 10.1016/s0168-8278(87)80060-0 3655309

[B22] Hsu HC Tseng HJ Lai PL Lee PH Peng SY Expression of p53 gene in 184 unifocal hepatocellular carcinomas: association with tumor growth and invasiveness Cancer Res 1993 53 4691 4694 8402647

[B23] Bachman KE Park BH Duel nature of TGF-beta signaling: tumor suppressor vs. tumor promoter Curr Opin Oncol 2005 17 49 54 10.1097/01.cco.0000143682.45316.ae 15608513

[B24] Pirruccello SJ LeBien TW The human B-cell-associated antigen CD24 is a single chain sialoglycoprotein J Immunolol 1986 136 3779 3784 2939133

[B25] Sagiv E Arber N The novel oncogene CD24 and its arising role in the carcinogenesis of the GI tract: from research to therapy Expert Rev Gastroenterol Hepatol 2008 2 125 133 10.1586/17474124.2.1.125 19072375

[B26] Fidler IJ Critical factors in the biology of human cancer metastasis: 28th G.H.A. Clowes memorial award lecture Cancer Res 1990 50 6130 6138 1698118

[B27] Baumann P Cremers N Kroese F Orend G Chiquet-Ehrismann R Uede T Yagita H Sleeman JP CD24 expression causes the acquisition of multiple cellular properties associated with tumor growth and metastasis Cancer Res 2005 65 10783 10793 10.1158/0008-5472.CAN-05-0619 16322224

[B28] Lim SC CD24 and human carcinoma: tumor biological aspects Biomed Pharmacother 2005 59 Suppl 2 351 354 16507407 10.1016/s0753-3322(05)80076-9

